# A Single Amino Acid of Human Immunodeficiency Virus Type 2 Capsid Protein Affects Conformation of Two External Loops and Viral Sensitivity to TRIM5α

**DOI:** 10.1371/journal.pone.0022779

**Published:** 2011-07-28

**Authors:** Tadashi Miyamoto, Masaru Yokoyama, Ken Kono, Tatsuo Shioda, Hironori Sato, Emi E. Nakayama

**Affiliations:** 1 Department of Viral infections, Research Institute for Microbial Diseases, Osaka University, Suita, Osaka, Japan; 2 Pathogen Genomics Center, National Institute of Infectious Diseases, Musashi Murayama, Tokyo, Japan; 3 Research Fellow of the Japan Society for the Promotion of Science, Tokyo, Japan; Chungbuk National University, Korea, Republic of

## Abstract

We previously reported that human immunodeficiency virus type 2 (HIV-2) carrying alanine or glutamine but not proline at position 120 of the capsid protein (CA) could grow in the presence of anti-viral factor TRIM5α of cynomolgus monkey (CM). To elucidate details of the interaction between the CA and TRIM5α, we generated mutant HIV-2 viruses, each carrying one of the remaining 17 possible amino acid residues, and examined their sensitivity to CM TRIM5α-mediated restriction. Results showed that hydrophobic residues or those with ring structures were associated with sensitivity, while those with small side chains or amide groups conferred resistance. Molecular dynamics simulation study revealed a structural basis for the differential TRIM5α sensitivities. The mutations at position 120 in the loop between helices 6 and 7 (L6/7) affected conformation of the neighboring loop between helices 4 and 5 (L4/5), and sensitive viruses had a common L4/5 conformation. In addition, the common L4/5 structures of the sensitive viruses were associated with a decreased probability of hydrogen bond formation between the 97th aspartic acid in L4/5 and the 119th arginine in L6/7. When we introduced aspartic acid-to-alanine substitution at position 97 (D97A) of the resistant virus carrying glutamine at position 120 to disrupt hydrogen bond formation, the resultant virus became moderately sensitive. Interestingly, the virus carrying glutamic acid at position 120 showed resistance, while its predicted L4/5 conformation was similar to those of sensitive viruses. The D97A substitution failed to alter the resistance of this particular virus, indicating that the 120th amino acid residue itself is also involved in sensitivity regardless of the L4/5 conformation. These results suggested that a hydrogen bond between the L4/5 and L6/7 modulates the overall structure of the exposed surface of the CA, but the amino acid residue at position 120 is also directly involved in CM TRIM5α recognition.

## Introduction

Human immunodeficiency virus type 1 (HIV-1) infects humans and chimpanzees but not Old World Monkeys (OWM) such as Rhesus monkey (Rh) and cynomolgus monkey (CM). This is attributed to a barrier in the host cell. In 2004, the screening of a Rh cDNA library identified TRIM5α as one of cellular antiviral factors [1]. TRIM5 is a member of the tripartite motif family containing RING, B-box and coiled-coil domains [2]. The alpha isoform of TRIM5 has an additional C-terminal PRYSPRY (B30.2) domain. Several studies have shown that sequence variation in variable regions of the PRYSPRY domain among different monkey species affects species-specific retrovirus infection [3]–[11].

Rh and CM TRIM5αs restrict HIV-1 but not simian immunodeficiency virus isolated from macaque (SIVmac) [1], [5], whereas African green monkey (AGM) TRIM5α inhibits both HIV-1 and SIVmac [5], [12]. Human TRIM5α only weakly restricts HIV-1, but potently restricts N-tropic murine leukemia virus (N-MLV) [11], [12].

Details of the molecular mechanism of retrovirus restriction by TRIM5α have been gradually elucidated by several groups. TRIM5α associates with the N-MLV capsid in detergent-stripped virions [13] or with an artificially constituted core structure composed of an HIV-1 capsid-nucleocapsid (CA-NC) fusion protein in a PRYSPRY domain-dependent manner [14], indicating that the target of TRIM5α is multimerized capsids. In addition, it was demonstrated that engagement of a restriction-sensitive retroviral core results in TRIM5α degradation by a proteasome-dependent pathway [15]. In the presence of proteasome inhibitors, virions complete reverse transcription and form functional pre-integration complexes, but 2-long terminal repeat circle formation and gene expression remain impaired [16], [17]. Recently, we have reported that AGM TRIM5α restricted SIVmac mainly via the proteasome-dependent pathway, whereas HIV-1 and HIV-2 restriction by AGM TRIM5α was both proteasome-dependent and proteasome-independent [18].

HIV-2 and SIVmac have very similar genomes [19], but vary in their ability to grow in the presence of TRIM5α from various species. SIVmac239 is resistant to Rh and CM TRIM5αs [1], [5], [8], whereas HIV-2 strains GH123 and ROD are sensitive to these TRIM5αs [5], [8], [20], [21]. We previously investigated the growth of eight different HIV-2 isolates in the presence of CM and human TRIM5αs and demonstrated that the growth of HIV-2 isolates carrying proline (P) at the 119th or 120th position of the capsid protein (CA) was inhibited by CM and human TRIM5αs, whereas the growth of those with either alanine (A) or glutamine (Q) was not affected by these TRIM5αs [20]. In a Caio cohort study in west Africa, it was demonstrated that subjects with a lower viral load more frequently carried a P at the 119th position of the CA, which corresponds to the 120th position of the GH123 CA, while non-proline residues at this position were more frequently observed in subjects with a high viral load [22], suggesting that TRIM5α controls viral replication in HIV-2-infected individuals.

The 120th amino acid is located in the loop between helices 6 and 7 (L6/7) [20]. Recently, we have succeeded in improving the replication of simian-tropic HIV-1 in CM cells by introducing the SIVmac L6/7 CA sequence [23]. In the present study, we generated mutant HIV-2 viruses each carrying one of the remaining 17 possible amino acid residues at the 120th position, and examined their susceptibilities to TRIM5α-mediated restriction in order to elucidate details of the interaction between HIV-2 CA and TRIM5α. Computer-assisted structural study showed that the mutations at position 120 in L6/7 affected conformation of the neighboring loop between helices 4 and 5 (L4/5).

## Results

### Amino acid residues at the 120th position of HIV-2 GH123 CA and viral susceptibility to CM TRIM5α

In a previous study, we reported that HIV-2 isolates carrying P at the 120th position of the CA were sensitive to CM and human TRIM5αs, whereas those with either A or Q were not [20]. In the Los Alamos sequence database, the amino acid residue at the 119th or 120th position of almost all HIV-2 CAs is P, A, Q or glycine (G). Therefore, we first generated mutant HIV-2 GH123 viruses carrying G at the 120th position (GH123/G) to investigate its effect on TRIM5α susceptibility.

**Figure 1:**
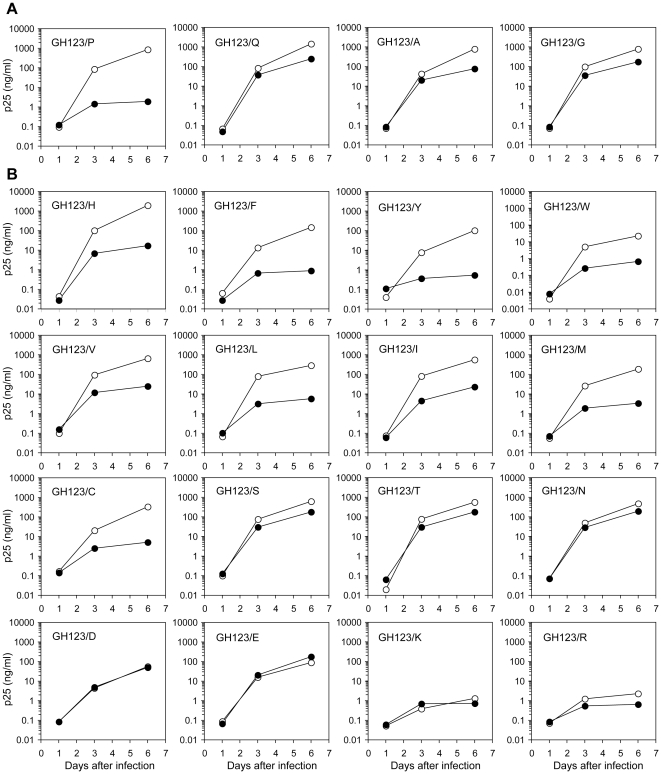
Growth of GH123 and its mutant viruses in the presence of CM TRIM5α. MT4 cells were infected with CM-TRIM5α-SeV (black circles) or CM-SPRY(–)-SeV (white circles) then superinfected with GH123 mutant viruses. Culture supernatants were periodically assayed for levels of virus capsid. Error bars show actual fluctuations between measurements of capsid in duplicate samples. A representative of two independent experiments is shown.

Equal amounts of p25 of mutant and wild type viruses were inoculated into the human T cell line MT4 expressing CM TRIM5α, and culture supernatants were periodically assayed for CA production. In agreement with the results of the previous study, wild type GH123 carrying P at the 120th position (GH123/P) was sensitive to CM TRIM5α since this virus failed to grow in the presence of CM TRIM5α. On the other hand, GH123/G as well as GH123/Q (glutamine) and GH123/A (alanine) were resistant to CM TRIM5α, since these viruses could grow in the presence of CM TRIM5α (Figure 1A).

To determine whether amino acid residues other than P, Q, A and G can occupy the 120th position of HIV-2 GH123 CA, and to elucidate further details of the interaction between the CA and TRIM5α, we generated 16 mutant GH123 viruses each carrying one of the remaining possible amino acid residues at the 120th position. As shown in Figure 1B, viruses with amino acid residues bearing a ring structure including aromatic groups, namely, histidine (GH123/H), phenylalanine (GH123/F), tyrosine (GH123/Y), tryptophan (GH123/W) and GH123/P were all sensitive to CM TRIM5α. Hydrophobic valine (GH123/V), leucine (GH123/L), and isoleucine (GH123/I) viruses as well as sulfated methionine (GH123/M) and cysteine (GH123/C) viruses were also sensitive.

In contrast, viruses with amino acid residues bearing hydroxyl or amide groups, namely, serine (GH123/S), threonine (GH123/T), glutamine (GH123/Q) and asparagine (GH123/N) were resistant to CM TRIM5α. Acidic aspartic acid (GH123/D) and glutamic acid (GH123/E) viruses were also resistant, although they grew to slightly lower titers than wild type GH123/P in the absence of CM TRIM5α. The replication of viruses with basic arginine (GH123/R) and lysine (GH123/K) was severely impaired and it was impossible to evaluate the effects of these residues on susceptibility to TRIM5α. Almost identical results were obtained when we inoculated equal amounts of reverse transcriptase of mutant and wild type GH123 (data not shown). Thus, the nature of the 120th amino acid residue greatly affects viral sensitivity to CM TRIM5α.

### CA processing is not affected by the 120th mutation

**Figure 2:**
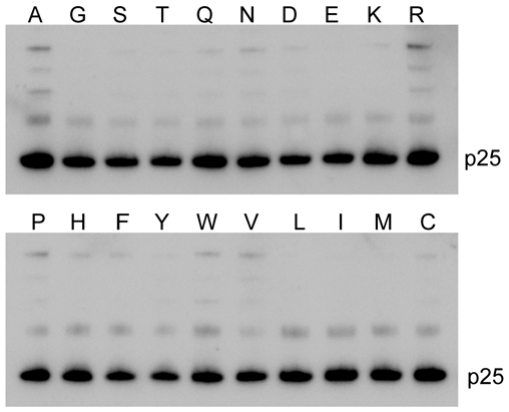
Western blot analysis of the CA in particles of GH123 and its mutant viruses. The viral particles of GH123 wild type and its mutant viruses were purified by ultracentrifugation through a 20% sucrose cushion. p25 capsid protein was visualized by western blotting (WB) using SIV-infected monkey serum.

To understand why GH123/R and GH123/K failed to replicate even in the absence of TRIM5α, we examined the Gag processing of mutant and wild type HIV-2 GH123 viruses using western blot analysis of viral particles. As shown in Figure 2, all mutant HIV-2 GH123 viruses produced viral particles with processed Gag proteins similar to the wild type virus. These results clearly exclude the possibility that the impaired replication of GH123/K and GH123/R viruses were due to inefficient processing of Gag precursors.

### The 118th position of SIVmac239 CA and viral susceptibility to CM TRIM5α

HIV-2, simian immunodeficiency virus isolated from sooty mangabey (SIVsm), and SIVmac have similar genomes [19]. SIVmac239 can replicate in the presence of CM TRIM5α [5] and contains Q at the 118th position, which corresponds to the 120th position of the GH123 CA. In our previous study, we reported that mutant SIVmac239 carrying P at the 118th position (SIVmac239/P) became sensitive to CM and human TRIM5αs [20]. In the present study, we examined whether other amino acid residues that conferred resistance (A, G) or sensitivity (valine, V) to CM TRIM5α or abolished viral replicative ability (arginine, R) on a GH123 background showed similar effects on viral sensitivity to CM TRIM5α on an SIVmac239 background.

**Figure 3:**
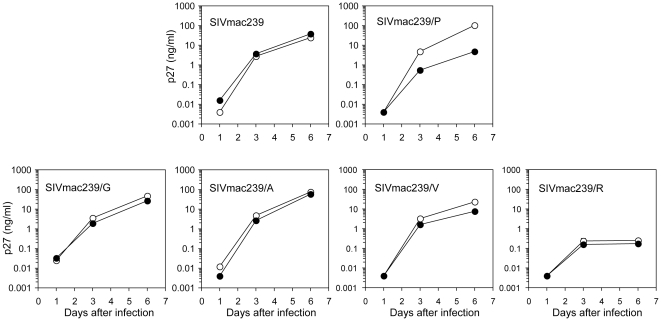
Growth of SIVmac239 and its mutant viruses in the presence of CM TRIM5α. MT4 cells were infected with CM-TRIM5α-SeV (black circles) or CM-SPRY(–)-SeV (white circles) then superinfected with SIVmac239 mutant viruses. Culture supernatants were periodically assayed for levels of virus capsid. Error bars show actual fluctuations between measurements of capsid in duplicate samples. A representative of three independent experiments is shown.

As shown in Figure 3, CM TRIM5α did not affect the replication of wild type SIVmac239 but inhibited SIVmac239/P, which is in agreement with the results of the previous study [20]. It should be noted, however, that the inhibitory effect of CM TRIM5α on SIVmac239/P was smaller than that on GH123/P, since SIVmac239/P demonstrated some growth even in the presence of CM TRIM5α. Newly generated SIVmac239 carrying alanine (SIVmac239/A) or glycine (SIVmac239/G) at the 118th position were unaffected by CM TRIM5α (Figure 3).

On the other hand, the mutant SIVmac239 carrying valine (SIVmac239/V) was only weakly inhibited by CM TRIM5α (Figure 3) to a lesser degree than the GH123/V. As shown in Figure 1, the inhibitory effect on GH123/V was also smaller than that on GH123/P even on a GH123 background. These results clearly indicate that single amino acid substitutions at the 118th position of the SIVmac239 CA had similar effects to those at the 120th position of GH123, although their impact was smaller in SIVmac239 than in the GH123 CA. Nevertheless, replication of mutant SIVmac239 carrying arginine (SIVmac239/R) was severely impaired, as with GH123/R.

### Molecular modeling and molecular dynamics (MD) simulations of the HIV-2 capsid N-terminal domain

The amino acid at position 120 is located in the L6/7 of the N-terminal domain of the CA. To obtain structural insights into the mechanisms by which this amino acid controls viral sensitivity to TRIM5α-mediated restriction, we conducted computer-assisted structural study of the N-terminal domain of the CA. With homology modeling and molecular dynamics (MD) simulation techniques, we constructed a series of initial structural models of the N-terminal half of the CA from CM TRIM5α-sensitive (GH123/P, GH123/F, GH123/H, and GH123/I) and CM TRIM5α-resistant (GH123/Q, GH123/A, GH123/N, and GH123/E) viruses. The initial models were then subjected to the MD simulation to analyze structural dynamics of the N-terminal domain of the CA in water environment. Average structures of individual CA mutants were obtained with 60,000 trajectories during 5–20 nanoseconds of MD simulations.

**Figure 4:**
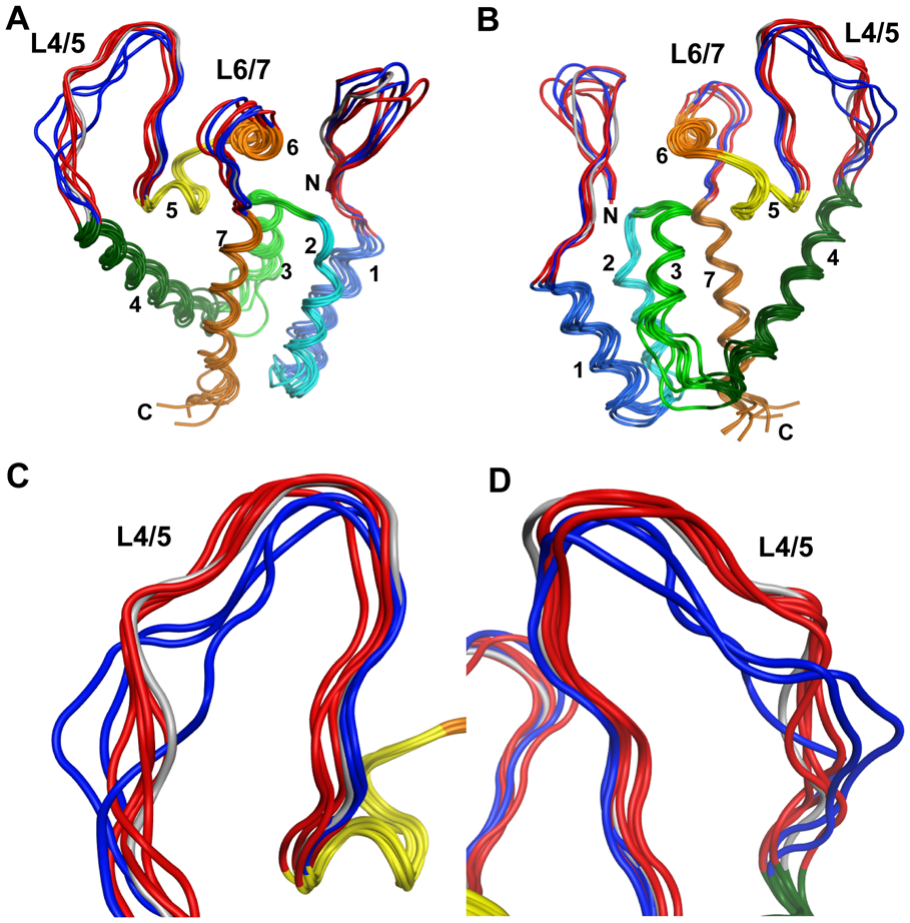
Structural models of the HIV-2 capsid N-terminal domain. Models were constructed by homology modeling and molecular dynamics simulations with the high-resolution X-ray crystal structure of the HIV-2 capsid N-terminal domain (PDB code: 2WLV [29]) as the starting structure. Averaged conformations of the overall structure of the N-terminal domain during 5–20 nanoseconds of MD simulations (A and B) and a close-up view around the L4/5 loop (C and D) are indicated. *N* and *C* indicate the amino termini and carboxyl termini, respectively; and the seven color-coded α-helices are labeled. Red and blue cartoons indicate the N-terminal loop, L4/5, and L6/7 of CM TRIM5α-sensitive (GH123/P, GH123/F, GH123/H and GH123/I) and CM TRIM5α-resistant (GH123/Q, GH123/A and GH123/N) viruses, respectively. Gray cartoons indicate the N-terminal loop, L4/5 and L6/7 of GH123/E in which the structures and biologic phenotypes are inconsistent. Models from two different angles are shown.

Comparisons of the average structures revealed that amino acid substitutions at position 120 could significantly influence the overall conformation of the exposed surface of the HIV-2 CA (Figure 4). Notably, the L4/5 of the mutant CAs are classified into two subgroups on the basis of their conformational similarities. These subgroups are primarily coincident with the two phenotypic subgroups based on viral sensitivities to CM TRIM5α, with the exception of mutant GH123/E (Figure 4, cartoon models indicated by gray). TRIM5α-sensitive viruses GH123/P, GH123/F, GH123/H and GH123/I showed almost identical L4/5 conformation (Figure 4, red models), while L4/5 of TRIM5α-resistant viruses GH123/Q, GH123/A and GH123/N were more variable (Figure 4, blue models). To confirm this, we performed additional modeling of TRIM5α-resistant viruses GH123/T and GH123/S. The results showed that L4/5 of GH123/T and GH123/S were also variable (data not shown).

**Figure 5:**
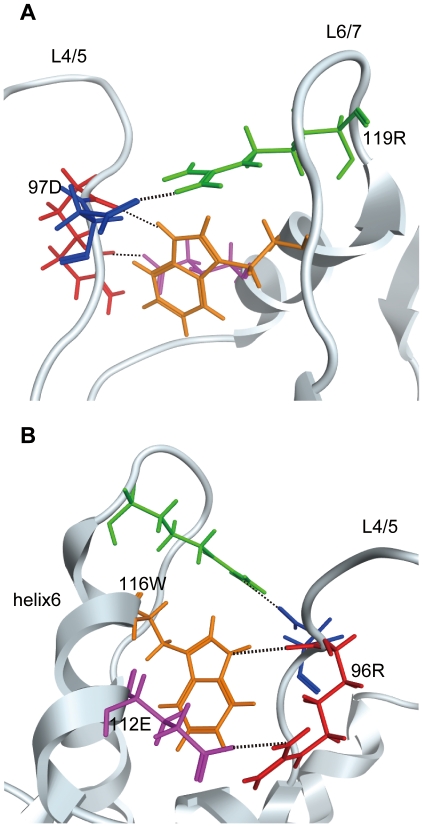
Hydrogen bond formation among L4/5, L6/7 and helix 6 of the HIV-2 CA. Close-up views of averaged structures of the N-terminal domain of the GH123/P CA during 5–20 nanoseconds of MD simulations are shown. Red, blue, purple, orange and green wireframes denote side chains of arginine at the 96th (96R), aspartic acid at the 97th (97D), glutamic acid at the 112th (112E), tryptophan at the 116th (116W) and arginine at the 119th (119R) positions, respectively. Dotted lines indicate hydrogen bonds visualized with MOE 2009. Models from two different angles are shown.

**Figure 6:**
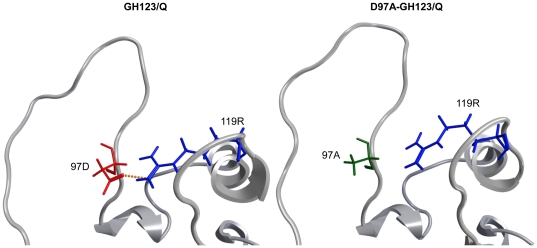
Lack of hydrogen bond formation between the 97th alanine and the 119th arginine of HIV-2 D97A-GH123/Q CA. Close-up views of averaged structures around the L4/5 loop of GH123/Q (left) and D97A-GH123/Q (right) during 5–20 nanoseconds of MD simulations are shown. Red, blue and green wireframes denote side chains of aspartic acid at the 97th (97D), arginine at the 119th (119R), and alanine at the 97th (97A) positions, respectively. A dotted line indicates a hydrogen bond.

**Figure 7:**
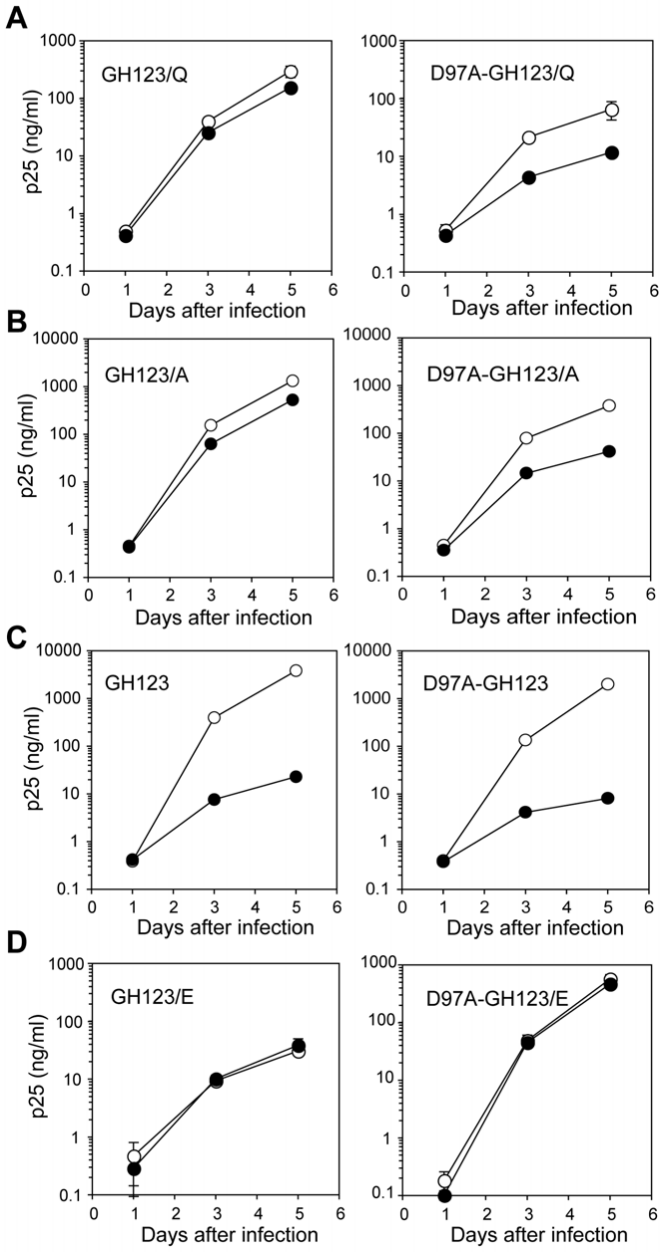
Effects of an aspartic acid-to-alanine substitution at the 97th position of the HIV-2 CA on viral growth in the presence or absence of CM TRIM5α. MT4 cells were infected with CM-TRIM5α-SeV (black circles) or CM-SPRY(–)-SeV (white circles) then superinfected with GH123 mutant viruses. Culture supernatants were periodically assayed for levels of viral capsid. Error bars show actual fluctuations between measurements of capsid in duplicate samples. A representative of three independent experiments is shown.

Furthermore, the MD simulation study revealed that the common L4/5 structures of the TRIM5α-sensitive viruses were associated with a reduced probability of hydrogen bond formation between the 97th aspartic acid (D) in L4/5 and the 119th arginine (R) in L6/7 compared with those of TRIM5α-resistant viruses except for GH123/E (Figure 5A and Table 1). We, therefore, hypothesized that the presence of the hydrogen bond between the 97th D in L4/5 and the 119th R in L6/7 disrupted the L4/5 conformation required for recognition by TRIM5α. To examine whether hydrogen bond formation between the 97th D and 119th R indeed affects the viral sensitivity to CM TRIM5α-mediated restriction, we introduced an alanine substitution at the 97th position of the TRIM5α-resistant viruses GH123/Q (D97A-GH123/Q) and GH123/A (D97A-GH123/A). The side chain of A at the 97th position would be too small to form a hydrogen bond with the 119th R, which was confirmed by MD simulation study of the D97A CA mutant of GH123/Q (Figure 6). As expected, the D97A substitution conferred moderate sensitivity to CM TRIM5α upon the resistant viruses GH123/Q and GH123/A (Figure 7A and 7B). In the case of TRIM5α-sensitive virus GH123/P, in which the probability of hydrogen bond formation between the 97th D and 119th R was predicted to be low (Table 1), the D97A substitution did not alter the viral senstivity to CM TRIM5α (Figure 7C). These data suggest that the conformation of L4/5, which is influenced by that of L6/7, participates in determining viral sensitivities to CM TRIM5α-mediated restriction. It should be noted, however, that the D97A substitution slightly impaired the replication of GH123/Q and GH123/A, as indicated by the titers of D97A-GH123/Q and D97A-GH123/A, which were apparently lower than those of GH123/Q and GH123/A at day 5 after infection even in the absence of TRIM5α (Figure 7A and 7B). Although we further tried to disrupt the hydrogen bond formation by introducing an alanine substitution at the 119th position, the resultant mutant viruses did not grow (data not shown). The arginine at the 119th position is highly conserved among different HIV-2 strains and may be essential for virus replication.

**Table 1:** The probability of forming a hydrogen bond between the 97th aspartic acid in L4/5 and the 119th arginine in L6/7 of the CA in 60,000 trajectories during 5–20 nanoseconds of MD simulations and the sensitivity phenotype.

120th amino acid	Frequency of hydrogen bond (%)	Sensitivity to CM TRIM5α
Pro (P)	44.6	Sensitive
Phe (F)	41.5	Sensitive
His (H)	42.99	Sensitive
Ile (I)	0	Sensitive
Ala (A)	64.47	Resistant
Gln (Q)	55.15	Resistant
Asn (N)	55.7	Resistant
Glu (E)	21.27	Resistant
Ser (S)	63.51	Resistant
Thr (T)	51.48	Resistant

**Figure 8:**
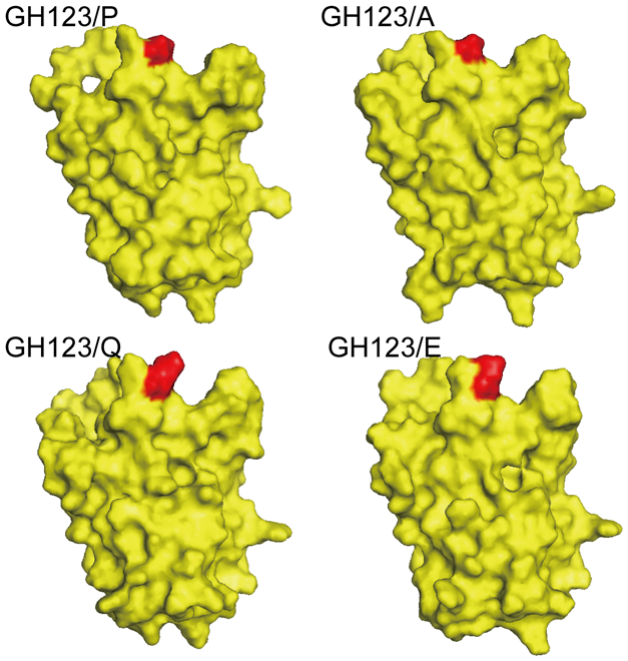
Surface structure of the HIV-2 capsid N-terminal domain. Surface structure of the GH123 and mutant GH123 CAs visualized with PyMOL. Red color indicates the 120th amino acid of the GH123 and mutant GH123 CAs.

In the case of the TRIM5α-resistant virus GH123/E (Figure 4, gray model), however, the conformation of L4/5 was similar to those of CM TRIM5α-sensitive viruses GH123/P, GH123/F, GH123/H and GH123/I (Figure 4, red models). The probability of hydrogen bond formation was also low in GH123/E, unlike that in the other resistant viruses GH123/Q, GH123/A and GH123/N (Table 1). Because GH123/E has a negatively charged amino acid E at the 120th position, we performed additional modeling of the CM TRIM5α-resistant virus with another negatively charged amino acid D (GH123/D). The results showed that the conformation of GH123/D L4/5 was also similar to those of CM TRIM5α-sensitive viruses (data not shown). Consistent with this, the possibility of hydrogen bond formation was low (21.27%) in GH123/D just as in GH123/E. It is possible that the presence of the negative charge at the 120th position prevented access of TRIM5α even though the L4/5 conformation was adequate for TRIM5α recognition. If our modeling of GH123/E L4/5 was correct, disruption of the hydrogen bond between the 97th D and 119th R would have little or no effect on the TRIM5α sensitivity of GH123/E. In fact, the D97A substitution failed to alter the resistant phenotype of GH123/E (Figure 7D), but did unexpectedly compensate the impaired replication of GH123/E (Figure 1B). These results indicate that the effect of D97A substitution depended upon the amino acid residue at the 120th position, and further supported the notion that the L6/7 itself was also involved in CM TRIM5α restriction. Consistent with this, the side chains of amino acid residues at the 120th position were exposed on the surface of the CA (Figure 8).

When these results are considered together, it is likely that the hydrogen bond between the L4/5 and L6/7 modulates the overall structure of the exposed surface of the CA and that both L4/5 and L6/7 are responsible for CA recognition by CM TRIM5α.

## Discussion

In the present study, we showed that a hydrogen bond between the 97th D and the 119th R of HIV-2 CA affected viral sensitivity to CM TRIM5α. TRIM5α-sensitive viruses showed a common L4/5 structure, but L6/7 was also important in CA recognition by TRIM5α.

Previously, we proposed that the configuration of HIV-2 CA L6/7 would affect viral sensitivity to CM TRIM5α on the basis of the results of homology modeling of the HIV-2 CA in which the 3-D structure of HIV-1 CA was used as a template [20]. In the present study, however, we performed intensive mutational analysis of the HIV-2 CA followed by more intensive computer-assisted structural analyses using the recently published 3-D structure of the HIV-2 CA and MD simulation, which provide information on structural dynamics of proteins in solution. Results of the present study revealed that alterations in the L4/5 conformation were more strongly associated with viral sensitivity to TRIM5α than those in the L6/7 configuration. Furthermore, the data on the MD simulation study disclosed that a hydrogen bond between the 97th D and the 119th R may be a critical modulator affecting the conformation of L4/5.

In the case of the HIV-1 CA, two hydrogen bonds were reported to form between R at the 229th position of Gag (R229) and E at the 245th position (E245), and between R229 and W at the 249th position (W249) [24]. These three amino acids were also found in the HIV-2 CA; and R229, E245 and W249 of the HIV-1 CA correspond to the 96th R, 112th E and the 116th W of the HIV-2 GH123 CA, respectively (Fig. 5B). The 112th E and 116th W are in the 6th helix of the CA, and the 96th R is adjacent to the 97th D in L4/5. In our HIV-2 CA models, these two hydrogen bonds were observed with a probability of more than 99.9%, regardless of the viral sensitivity to TRIM5α. Therefore, TRIM5α-resistant viruses are likely to have three hydrogen bonds at the base of L4/5, whereas those sensitive to TRIM5α have two hydrogen bonds there. It is possible that reduced structural flexibility of the base of loop causes the upper loop structure to collapse more easily. Thus, the number of the hydrogen bonds may affect the flexibility of the base of L4/5 and the maintenance of the binding surface for TRIM5α, which is formed at least partly by L4/5. As a result, the viral sensitivity to TRIM5α changes.

In the CA sequences of HIV-2 and SIVmac in the Los Alamos Database, the 97th position was always occupied by acidic D or E, and the 119th position was always occupied by R. In the case of HIV-1 or simian immunodeficiency virus isolated from the chimpanzee (SIVcpz), however, the 119th position was occupied by variable amino acid residues, while the 97th position was always occupied by acidic D or E. It should be noted that a hydrogen bond between the 97th and 119th amino acid residues was never observed in the HIV-1 CA (data not shown). Those differences may contribute to the increased sensitivity of HIV-1 to OWM TRIM5α compared with HIV-2 strains.

Although our data showed a clear correlation between viral sensitivity to TRIM5α and the conformation of CA L4/5, there was one exception. The conformation of L4/5 in GH123/E was almost identical to those of TRIM5α-sensitive viruses, but GH123/E was highly resistant to CM TRIM5α. Furthermore, disruption of the hydrogen bond between the 97th D and the 119th R by substitution of D97A did not alter the resistant phenotype of GH123/E at all. These results suggested that the 120th amino acid residue of the HIV-2 GH123 CA itself is also involved in CM TRIM5α sensitivity independently from the L4/5 conformation. This view was also supported by our present observation that disruption of the hydrogen bond between the 97th D and the 119th R conferred only moderate sensitivity to CM TRIM5α upon another resistant virus GH123/Q (Figure 7).

Replication of GH123/E or GH123/D was slightly impaired (Figure 1B), but this impairment was compensated by the D97A substitution in GH123/E (Figure 7D). On the other hand, replication of GH123/Q was almost comparable to that of GH123 (Figure 1A); but the D97A substitution slightly impaired its replicative capability (Figure 7A). It should be also mentioned here that the viruses with basic residues at the 120th position, GH123/R and GH123/K, scarcely grew (Figure 1B). These results suggest that certain optimum levels of charge are required at the L4/5 and L6/7 for efficient viral replication. At present, it is unclear why those charge differences affect the growth capability of the virus; but it is possible that the charge difference affects the accessibility to unknown host factor(s) involved in uncoating.

HIV-2 closely resembles SIVsm, which is thought to have entered the human population on at least eight separate occasions [19]. Almost all SIV isolates from the Los Alamos Database contain glutamine at the position corresponding to the 119th or 120th position of the HIV-2 CA in the presence of strong OWM TRIM5α pressure. After entry of SIVsm into the human population, which lacks OWM TRIM5α pressure, some viruses were presumably forced to change glutamine to proline by mutating the second nucleotide of the codon. This change may have been driven by specific immune responses against the HIV-2 CA. Similarly, alanine viruses may have evolved from the proline virus after transmission to individuals lacking such responses by changing the first nucleotide of the codon in order to become more resistant to human TRIM5α. Glycine viruses may have further evolved from the alanine virus by changing the second nucleotide of the codon. However, it is unclear why serine, histidine, threonine and leucine viruses have not been identified despite their nearly normal levels of growth. It is possible that certain human immune responses prevented their emergence.

In a sharp contrast to CM TRIM5α, Rh TRIM5α could restrict both CM TRIM5α-sensitive and -resistant HIV-2 strains [8]. SIVmac239 is resistant to Rh TRIM5α, but chimeric SIVmac239 with L4/5 of HIV-2 strains GH123 [25] or ROD [21] were efficiently restricted by Rh TRIM5α. Therefore, the L4/5 of HIV-2 CA is also a critical determinant for Rh TRIM5α-mediated restriction. In the present study, we have shown that CM TRIM5α-sensitive HIV-2 viruses have a specific structure in the L4/5 of the CA. However, the 3-D structure of Rh and CM TRIM5α remains unsolved. To elucidate the more detailed molecular mechanism of the interaction between TRIM5α and the CA, structural information about TRIM5α is essential. A docking study based on such information is likely to shed light on the antiviral mechanism of TRIM5α.

In summary, we showed that a hydrogen bond between the 97th D and the 119th R of HIV-2 CA affected viral sensitivity to CM TRIM5α and that both L4/5 and L6/7 are responsible for CA recognition by CM TRIM5α.

## Methods

### Cell cultures

293T cells were maintained in Dulbecco's Modified Eagle medium, and HeLa cells were maintained in Minimum Essential Medium. The human T-cell line MT4 was maintained in RPMI medium. All media were supplemented with 10% fetal bovine serum and 1% penicillin-streptomycin.

### Plasmid construction

Mutant HIV-2 GH123 or SIVmac239 viruses were generated by site-directed mutagenesis. Infectious viruses were prepared by transfection of 293T cells with resultant proviral DNA clones. The viral titer was determined by measuring p25 or p27 with a RetroTek antigen ELISA kit (ZeptoMetrix, Buffalo, NY).

Construction of recombinant Sendai viruses (SeV) expressing C-terminally HA-tagged CM TRIM5α (CM-TRIM5α-SeV) and CM-TRIM5α lacking the PRYSPRY domain (CM-SPRY(–)-SeV) were described previously [5], [20].

### Viral infection

MT4 cells (1×10^5^) were infected with SeV expressing each of the TRIM5αs at a multiplicity of infection of 10 plaque-forming units per cell and incubated at 37°C for 9 h. Cells were then superinfected with 20 ng of p25 of HIV-2 GH123 derivatives or with 40 ng of p27 SIVmac239 derivatives. The culture supernatants were collected periodically, and the level of p25 or p27 was measured with a RetroTek antigen ELISA kit (ZeptoMetrix).

### Viral particle purification and western blotting

The culture supernatant of 293T cells transfected with plasmids encoding HIV-2 GH123 and GH123 mutants were clarified by low-speed centrifugation. The resultant supernatants (10 ml) were layered onto a 2 ml cushion of 20% sucrose and centrifuged at 35,000 rpm for 2 h at 4°C in a Beckman SW41 rotor. Pelleted viral particles were resuspended in PBS. Lysates were normalized based on p25 antigen concentrations and were analyzed by western blotting with the SIV-infected monkey serum.

### Molecular modeling and MD simulation

We used MD simulations [26] to analyze structural dynamics of the HIV-2 CA N-terminal domain. First, initial CA structures for MD simulation were constructed by homology modeling [27] using the Molecular Operating Environment, MOE 2008.1002 (Chemical Computing Group Inc., Montreal, Quebec, Canada) as described [20], [28]. We used the high-resolution crystal structure of the HIV-2 CA N-terminal domain at a resolution of 1.25 Å (PDB code: 2WLV [29]) as the modeling template. Structural dynamics of these HIV-2 CA models in water environment were analyzed using MD simulations with the SANDER module in the AMBER 9 program package [30] and the AMBER99SB force field [31] with the TIP3P water model. Bond lengths involving hydrogen were constrained with SHAKE [32] and the time step for all MD simulations was set to 2 fs. After heating calculations for 20 ps to 310 K using the NVT ensemble, the simulations were executed using the NPT ensemble at 1 atm and at 310 K for 20 ns. Hydration analyses were performed using the ptraj module in AMBER. A maximum cutoff angle of 120.0° and cutoff length of 3.5 Å were used in hydrogen bond definitions. The surface structure of CA is visualized with PyMOL 1.2r1 (The PyMOL Molecular Graphics System, http://pymol.sourceforge.net/).
